# Extraction decision and identification of treatment predictors in Class I malocclusions

**DOI:** 10.1186/2196-1042-14-47

**Published:** 2013-11-19

**Authors:** Dimitrios Konstantonis, Chrysi Anthopoulou, Margarita Makou

**Affiliations:** Department of Orthodontics, School of Dentistry, University of Athens, Athens, 11527 Greece

**Keywords:** Class I, Extractions, Extraction rate, Treatment predictors, Discriminant analysis

## Abstract

**Background:**

The extraction rate in orthodontics varies throughout the years. While the extraction decision is easily made or excluded in clear-cut cases, it still remains controversial what makes an orthodontist decide to extract in borderline cases. The aim of this retrospective study was to identify the percentage of extraction cases in a large group of Class I malocclusions and to clarify which variables contributed most to the extraction decision.

**Methods:**

The sample consisted of 542 randomly selected records of Class I patients treated in a university graduate program and in five private orthodontic offices. Of these patients, 331 were female and 211 male. The mean age was 14.55 (standard deviation (SD) 5.36) for the non-extraction group and 14.52 (SD 4.86) for the extraction group. The extensive series of 32 linear and angular measurements derived from the cephalometric analysis and the dental casts, along with the variables of age and gender, fueled a stepwise discriminant analysis.

**Results:**

The percentage of the patients treated with four first premolar extractions was 26.8%. The results showed that the variables of lower crowding, lower lip to E-plane, upper crowding, and overjet accounted most for the decision to extract at a very significant level (Sig. 0.000). The discriminant analysis assigned a classification power of 83.9% to the predictive model (*p* < 0.0001). Fisher's linear discriminant functions provided a mathematical model, according to which any case can be classified into the adequate treatment group.

**Conclusions:**

In a large contemporary sample of 542 Class I patients, the extraction rate was 26.8%. The most important measurements when the orthodontist decides extractions in Class I cases are lower crowding, lower lip to E-plane, upper crowding, and overjet. In clinical orthodontic practice, the findings facilitate treatment by providing evidence-based treatment predictors for Class I malocclusions.

## Background

In treating a Class I malocclusion by means of comprehensive orthodontics, there are two main therapeutic approaches: extraction and non-extraction. Extractions are routinely used to address dental crowding and reduce protrusion of the teeth and the overlying soft tissue. The alternative treatment is expansion of the arches. The extraction rate in orthodontics shows strong variations depending on the decade and socioeconomic factors. In the 1950s, 10% of the cases were treated with extractions whereas in the following decade, the percentage climbed up to 50% until the 1980s when it dropped to the contemporary number of 30% [1–5].

In diagnosing and treatment planning a case, the orthodontist examines a series of variables that lead him to his final decision. These variables are the measurements of the cephalometric analysis and the models along with the age and sex of the patient. Other factors like periodontal condition, restorations, and congenitally missing or extracted teeth also have an impact on the decision. After taking all of the above factors into consideration, the treatment plan is established and the need for or not for extractions is justified [6, 7].

The knowledge of the variables which account for favoring one therapeutic approach over the other will help expedite the decision making and will serve to establish treatment predictors. The numerical value of these variables will also be a valuable tool when diagnosing a Class I case. In order to identify which variables have an impact on the orthodontist's decision whether to extract or not, it is necessary to know in which characteristics patients treated in one way tend to differ from those treated in another way. The characteristics of the patient that lead a clinician to a given treatment decision are known as confounding variables. Discriminant analysis is the ideal statistical multivariate technique that deals simultaneously with large numbers of confounding variables [8, 9]. Discriminant analysis has been proven to mimic effectively the decision process of experienced orthodontists [10–13]. The main reason that this analysis is employed in current orthodontic retrospective surveys is its ability to predict group membership, to identify patients who could belong to either group (borderline), and to establish treatment predictors [14–22].

The aim of this study was to identify the percentage of cases treated with four first premolar extractions in a large sample of Class I patients and to suggest which of the orthodontic measurements are the most important in leading an orthodontist to his treatment decision.

## Methods

### Data collection

The records of 542 Class I patients were randomly selected from a university graduate orthodontic clinic (University of Athens, Greece) and five private orthodontic offices. It was decided to gather records from a university clinic where there is a substantial number of different supervisors and residents and also from five different private offices in order to eliminate selection or proficiency bias attempting thus to reflect contemporary treatment philosophy regarding extraction treatment of Class I malocclusions.

All patients were Caucasian males and females with a full complement of teeth (excluding the third molars) who presented with a Class I dental and skeletal malocclusion. They had no history of any cleft, dentofacial deformity, or syndrome, and they neither had ever received any previous orthodontic treatment. Of the patients, 397 were treated non-extraction whereas 145 were treated by extraction of the four first premolars, and 331 were female and 211 were male. Of the female patients, 243 (61.2%) were treated non-extraction whereas 88 (60.7%) received extraction treatment. Of the male patients, 154 (38.8%) were treated non-extraction and 57 (39.3%) received extraction treatment (Table 1). The mean patient age was 14.55 (standard deviation (SD) 5.36) and 14.52 (SD 4.86) for the non-extraction and the extraction group, respectively.

**Table 1:** **Treatment-gender cross-tabulation and their association**

Treatment		Male	Female	Total
Non-extraction	Count	154	243	397
	% within treatment	38.8	61.2	100.0
	% within gender	73.0	73.4	73.2
Extraction	Count	57	88	145
	% within treatment	39.3	60.7	100.0
	% within gender	27.0	26.6	26.8
Total	Count	211	331	542
	% within treatment	38.9	61.1	100.0
	% within gender	100.0	100.0	100.0

Fisher's exact test: exact significance (two-sided) = 0.921.

In an attempt to have the exact representation of the dental, skeletal, and soft tissue data the clinician considered and analyzed before his treatment decision, the measurements were all gathered from the patients' initial charts. Twenty-six cephalometric and six model measurements along with the variables of age and gender were subjected into statistical analysis (Table 2). All cephalometric radiographs were taken in natural head position, and the cephalometric analyses were performed using Viewbox 4.0.1.7 (dHAL Software, Kifissia, Greece).

**Table 2:** **Cephalometric and model measurements and demographic variables**

	Variables	Characteristics
Cephalometric measurements		
1	SNA	Maxillary position
2	SNB	Mandibular position
3	ANB	Maxillo-mandibular relationship
4	U1-SN	Upper incisor inclination
5	U1-NA	Upper incisor inclination
6	NSGn	Mandibular size/position
7	FMIA	Lower incisor inclination in relation to FH
8	IMPA	Lower incisor inclination in relation to MP
9	FMA	Facial height/orientation of the mandible
10	L1-NB	Lower incisor inclination in relation to NB
11	U1-L1	Upper-lower incisor relationship
12	SN-PP	Palatal position/cant
13	SN-OP	Occlusal plane cant/position
14	Z angle	Profile convexity
15	PNS-A	Maxillary size
16	U1-NA	Upper incisor position and inclination
17	L1-NB	Lower incisor position and inclination
18	L1-A Pg	Lower incisor position
19	Pg-NB	Bony chin size
20	WITS	Maxillo-mandibular relationship
21	N-Me	Total face height
22	N-ANS	Upper face height
23	ANS-Me	Lower face height
24	LL-E-plane	Lower lip protrusion
25	S-Go	Mandibular position
26	S-Ar	Mandibular position
Model measurements		
27	Overbite	
28	Overjet	
29	Upper crowding	
30	Lower crowding	
31	Upper midline deviation	
32	Lower midline deviation	
Demographic variables		
33	Age	
34	Gender	

### Statistical analysis

Statistical analysis was carried out using SPSS version 19.0 (SPSS Inc., Chicago, IL, USA) and Minitab 16 software and included the following steps: means, standard deviations, and *p* values (*t* test for independent samples) for all variables of both groups were calculated (Table 3). Fisher's exact test was also performed to test the possible association between gender and treatment choice. The data consisting of these 34 independent variables were then subjected to a stepwise discriminant analysis. In the stepwise method, which is indicated when dealing with a large number of independent variables, the variables enter the discriminant function one at a time on the basis of their discriminating power. Initially, the single best discriminating variable is chosen. The first variable is therefore paired to each of the other independent variables, and a second variable is chosen. The second variable is the best one to improve the discriminating power of the function along with the first variable. In a similar manner, other independent variables also enter the function. As additional variables are included, some of the previous ones may be removed if the information they contain about group differences is available in a combination of the other included variables [23]. In this model, the variables enter in a stepwise fashion using Wilk's lambda criterion. It is noted that the criteria for the removal of a variable are stricter than the corresponding entry criteria. Additionally, there is no guarantee that the final model has included all significant variables or that it has not implemented some non-significant ones. Nevertheless, given the number of independent variables, the stepwise method is considered the most appropriate choice.

**Table 3:** **Descriptive statistics**

		Non-extraction	Extraction	***p*** value^a^
Cephalometric variables				
SNA	Mean	81.76	81.26	0.184
	SD	3.93	3.79	
SNB	Mean	78.54	77.11	0.000
	SD	3.65	3.54	
ANB	Mean	3.16	4.08	0.000
	SD	2.27	2.21	
U1-SN	Mean	104.12	103.23	0.215
	SD	7.26	7.71	
U1-NA (1)	Mean	22.15	21.72	0.535
	SD	7.00	7.62	
NSGn	Mean	67.13	68.88	0.000
	SD	4.09	3.71	
FMIA	Mean	61.95	60.32	0.022
	SD	7.55	6.71	
IMPA	Mean	92.38	92.58	0.763
	SD	5.99	5.51	
FMA	Mean	25.60	27.05	0.011
	SD	397	145	
L1-NB (1)	Mean	24.99	25.72	0.258
	SD	6.59	6.66	
U1-L1	Mean	129.48	128.30	0.257
	SD	10.51	11.26	
SN-PP	Mean	7.09	7.25	0.631
	SD	3.52	3.11	
SN-OP	Mean	16.27	17.72	0.001
	SD	4.58	4.63	
Z angle	Mean	75.05	71.79	0.000
	SD	7.04	6.39	
PNS-A	Mean	48.97	49.01	0.939
	SD	4.48	5.26	
U1-NA (2)	Mean	3.97	4.18	0.479
	SD	2.56	3.12	
L1-NB (2)	Mean	4.34	5.18	0.000
	SD	2.39	2.59	
L1-A Pg	Mean	1.92	2.38	0.056
	SD	2.44	2.63	
Pg-NB	Mean	1.43	0.94	0.007
	SD	1.89	1.66	
WITS	Mean	-0.15	0.58	0.023
	SD	2.82	3.41	
N-Me	Mean	114.67	116.74	0.073
	SD	10.15	12.41	
N-ANS	Mean	51.03	51.48	0.380
	SD	5.12	5.74	
ANS-Me	Mean	65.84	67.46	0.030
	SD	6.39	8.03	
LL-E-plane	Mean	-1.72	-0.22	0.000
	SD	3.12	2.90	
S-Go	Mean	72.93	72.31	0.449
	SD	8.08	9.54	
S-Ar	Mean	33.98	33.54	0.297
	SD	4.36	4.41	
Model variables				
Overbite	Mean	3.14	3.53	0.033
	SD	1.93	1.71	
Overjet	Mean	2.85	3.45	0.004
	SD	1.94	2.21	
Upper crowding	Mean	-1.16	-5.00	0.000
	SD	3.79	3.79	
Lower crowding	Mean	-1.98	-6.63	0.000
	SD	3.17	3.60	
Upper midline deviation	Mean	0.06	0.32	0.034
	SD	1.00	1.32	
Lower midline	Mean	0.00	-0.09	0.573
	SD	1.19	1.60	
Demographic variables				
Age	Mean	14.55	14.52	0.948
	SD	5.36	4.86	

^a^ *t* test for independent samples.

Because of the difference in measuring units (degrees and millimeters for angular and linear measurements, respectively), the ‘standardized canonical discriminant function coefficients’ were calculated. The Bayes probabilities were then employed in order to identify the classification of the cases according to the predictive model. Additionally, Press's *Q* statistic was applied to test if the ‘hit ratio of the model’ is significantly better than chance. Finally, Fisher's linear discriminant function coefficients provided an equation, according to which every case could be classified.

## Results

Gender was not a consideration in treatment planning since the same percentage of females (26.6%) and males (27%) received extraction treatment. This observation was also verified by Fisher's exact test, which compared treatment choice with patient's gender (*p* < 0.921; Table 1).

Analyzing the data gathered from the sample, the discriminant function incorporated four significant (*p* < 0.000) discriminating variables which, in descending order of importance (based on the magnitude of their standardized canonical function coefficients), were lower crowding (0.728), lower lip to E-plane (-0.407), upper crowding (0.347), and overjet (-0.219). The independent variable of lower crowding accounted most for the formation of the specific treatment decision (extraction versus non-extraction) and was the one variable that possessed unique discriminating power. The mean values of the four discriminating variables are listed in Table 4. A summary of the stepwise discriminant analysis with the four incorporated discriminating variables is listed in Table 5. It must be noted that each one of the variables entered at a high level of significance (Sig. 0.000).

**Table 4:** **Descriptive statistics for the discriminating variables**

	Treatment	***N***	Mean	SD	***p*** value^a^
Lower crowding	Non-extraction	397	-1.98	3.17	0.000
	Extraction	145	-6.63	3.60	
LL-E-plane	Non-extraction	397	-1.72	3.12	0.000
	Extraction	145	-0.22	2.90	
Upper crowding	Non-extraction	397	-1.16	3.78	0.000
	Extraction	145	-5.00	3.79	
Overjet	Non-extraction	397	2.85	1.94	0.004
	Extraction	145	3.45	2.21	

^a^ *t* test for independent samples. LL, lower lip.

**Table 5:** **Stepwise discriminant analysis**

Step	Variable	Wilk's lambda	Standardized canonical coefficient	Significance
1	Lower crowding	0.718	-0.728	0.000
2	LL-E-plane	0.673	-0.407	0.000
3	Upper crowding	0.662	0.347	0.000
4	Overjet	0.653	-0.219	0.000

The value of Wilk's lambda (0.653) indicated that the canonical discriminant function achieved a significant (Sig. 0.000) degree of discrimination between the two different treatment group centroids (Table 6). The discriminant analysis was successful into assigning a classification power of 83.9% (overall hit ratio) to the predictive model. Additionally, 83.8% of the cross-validated cases were also correctly classified. The classification results (Table 7) revealed that from the 397 non-extraction cases, 377 were predicted as such and 20 as extraction. From the 145 extraction cases, 78 were predicted as extraction and 67 as non-extraction. According to Press's *Q* statistic, the analysis was proven to be better than the maximum chance criterion (MCC) into assigning treatment group (*p* < 0.0001; Table 8).

**Table 6:** **Wilk's lambda**

Test of function	Wilk's lambda	Chi-square	***df***	Significance
1	0.653	229.693	4	0.000

**Table 7:** **Classification results**

		Treatment	Predicted group membership		Total
			Non-extraction	Extraction	
Original	Count	Non-extraction	377	20	397
		Extraction	67	78	145
	%	Non-extraction	95.0	5.0	100.0
		Extraction	46.2	53.8	100.0
Cross-validated^a^	Count	Non-extraction	376	21	397
		Extraction	67	78	145
	%	Non-extraction	94.7	5.3	100.0
		Extraction	46.2	53.8	100.0

^a^Cross-validation is done only for those cases in the analysis. In cross-validation, each case is classified by the functions derived from all cases other than that case; 83.9% of original and 83.8% of cross-validated grouped cases were correctly classified. The proportional by chance accuracy rate was computed by squaring and summing the proportion of cases in each group from the table of prior probabilities for groups (0.73^2^ + 0.27^2^ = 0.607). A 25% increase over this would require that our cross-validated accuracy be 75.8% (1.25 × 60.7 % = 75.8%). The cross-validated accuracy rate computed by SPSS was 83.8% which was greater than the proportional by chance accuracy criteria of 75.8%.

**Table 8:** **Press's** ***Q*** **statistic**

	Hit ratios
MCC	73.2%
Discriminant model	83.9%
*Q* test (*p* value)	249.8598 (<0.0001)

The discriminant model proved to be more powerful than the maximum chance criterion into correctly classifying the cases.

**Figure 1:**
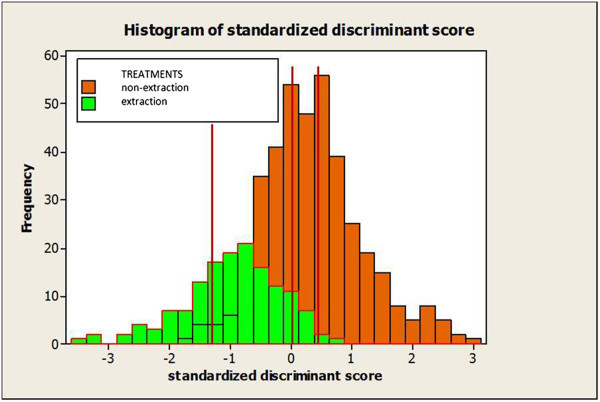
**Histogram of standardized discriminant scores.** The red vertical lines indicate the optimal cutoff point at -0.0001 and the group centroids at 0.440 for the non-extraction and -1.205 for the extraction group.

**Figure 2:**
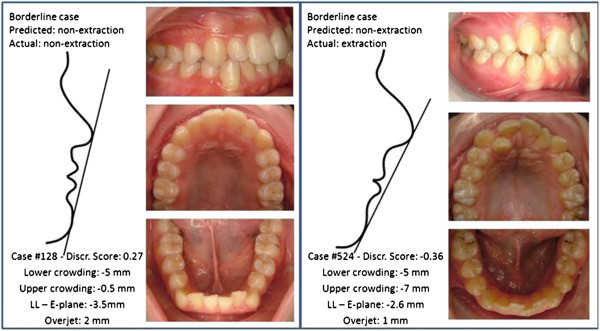
**Discriminating variables for two borderline cases.** According to the discriminant analysis, case #128 was correctly classified whereas case #524 was misclassified.

**Figure 3:**
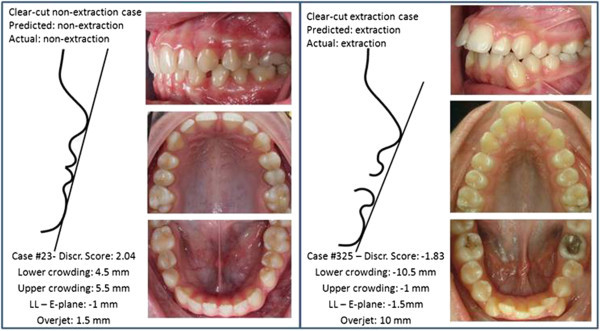
**Discriminating variables for two clear-cut cases.** According to the discriminant analysis, both cases were correctly classified.

The discriminant analysis provided a discriminant score for each single patient. Patients with negative scores were most likely to be extraction cases and those with a positive score probably received non-extraction treatment. The range of the discriminant scores was -3.4889 to 3.0687. The group centroids which represent the mean of the discriminant scores were 0.440 for the non-extraction and -1.205 for the extraction group (Figure 1). The optimal cutting score value was -0.0001 (weighted mean of the two centroids). Most of the misclassified cases were around the cutting score. That was an indication that the borderline spectrum was correctly identified. Cases with higher discriminant scores were classified mostly correctly, thus representing the clear-cut extraction or non-extraction cases. In Figures 2 and 3, four representative cases of clear-cut and borderline spectra are shown.

**Figure 4:**
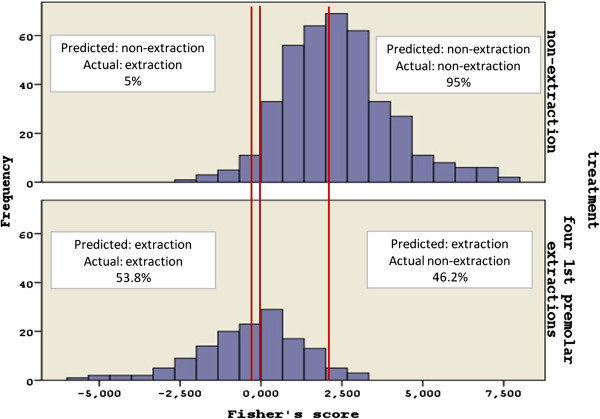
**Histogram of Fisher's scores.** The red vertical lines indicate the optimal cutoff point at 0 and the group centroids at 2.360 for the non-extraction and -0.347 for the extraction group.

An alternative way of classifying the cases is by using Fisher's criterion. This method provides a mathematical equation, according to which classification is achieved. In the present study, the equation resulted as follows:

If *F* > 0, then the case is classified into the non-extraction and if *F* < 0, into the extraction group. The closest the score is to the group centroids (-0.347 for the extraction and 2.360 for the non-extraction), the more likely the case is to be treated decisively by either extraction or non-extraction. Still, when the score is around 0, the case is considered borderline (Figure 4).

## Discussion

In orthodontics, extractions have been traditionally highly debated and their percentage has displayed considerable variation throughout the years depending on treatment trends and other various factors. In the present study, the extraction rate of Class I malocclusions was 26.8%, therefore being in relative accordance with similar findings of other authors.

According to the study carried out by Proffit [4] at the University of North Carolina in the 1950s, only 10% of the cases were treated with four first premolar extractions. The following decade, the percentage attained its peak with 50% and remained there until the 1980s when it started decreasing. The author attributed the decrease in extraction rates to the lack of evidence in the literature regarding treatment stability after extractions, as well as to the non-evidence-based theory of extraction association to TMJ dysfunction. Numerous studies suggest that biotechnology innovations along with the tendency for fuller lips bring the extraction rate up to 30%, hence reaching the level of the early 1990s [2, 3, 5].

Orthodontists traditionally follow a specific diagnostic process which helps them gain confidence into decision making. Parts of this process are the cephalometric analysis, the study of diagnostic dental casts, and the consideration of other parameters such as age, gender, and teeth with poor prognosis. The decision seems easier to make when addressing a clear-cut rather than a borderline case. The discriminant analysis was successful into correctly classifying 83.9% of the cases; 95% of the non-extraction and 53.8% of the extraction cases were correctly classified. Most of the misclassified cases were in the borderline spectrum around the cutting score. In borderline cases, the decision to extract depends solely on the orthodontist the patients happened to visit. The 46.2% of the misclassified extraction cases indicates the reluctance of the orthodontist to extract in a borderline case. In this study, we tried to quantify clinicians' favorite parameters, according to which his decision about extractions is made.

The discriminant analysis incorporated four variables that were unique in their ability to discriminate between the two different treatment approaches. The first variable which entered the function was the measurement of lower crowding. Indeed, the clinicians base a big part of their treatment decision on the amount of crowding the patient exhibits. This variable was found to be of paramount importance in similar studies conducted in the early 1990s [10, 11, 13]. The second most important variable of lower lip to E-plane is an indication of the patient's profile. A widely used measurement which is the distance of the lower lip to the E-plane as suggested by Ricketts [24] still remains a very prevalent tool when diagnosing a case. When the lips show inadequate projection, the orthodontist is quite reluctant to extract, but when they exceed the E-plane, extractions are easier to decide. This result came to verify the importance facial aesthetics have for the vast majority of orthodontists upon treatment planning [25–31]. Upper crowding was found to be the third important variable which could possibly lead to an extraction decision. Furthermore, the fourth discriminating variable of overjet in Class I cases constitutes an indication of teeth and soft tissue projection, thus playing an important role in balanced dental and facial aesthetics. Excessive overjet is usually noted in dentoalveolar bimaxillary protrusion cases which they are routinely addressed with removal of four first premolars. It should also be noted that in Class I cases, increased overjet occurs when severe lower crowding is present.

It is interesting that all the variables represent linear measurements routinely obtained by the orthodontist upon clinical examination. The amount and position of the lips in relation to the face can also be estimated upon clinical screening, yet clinical appraisals were not included in the present study. Surprisingly, the orientation of the lower incisor to the basal bone or the face as appraised in various angles like IMPA or FMIA was not included into the discriminating variables. In similar studies, the lower incisor angle was found to be an important variable with the ability to discriminate between the two treatment modalities [10, 11, 13, 31]. Is orthodontics drifting away from cephalometrics as a valuable aid in diagnosis? It remains to be seen.

There are probably other popular measurements of morphological characteristics on which clinicians base their treatment decision, but these four were detected by the discriminant analysis as the most important ones when deciding extractions. These findings apply to the sample they were derived from which was 542 Caucasian patients of European descent and will possibly vary if the research is repeated in different populations treated by other orthodontists. The equation derived from the discriminant analysis is a useful adjunct to consult when in doubt regarding extractions in Class I cases.

## Conclusions

In a large contemporary sample of 542 Class I patients treated in Greece, the extraction rate was 26.8%. According to the discriminant analysis, when deciding extractions to address Class I cases, the orthodontist mainly considers the measurements of lower crowding, lower lip to E-plane, upper crowding, and overjet. The employment of a simple mathematical model which includes four ‘key’ orthodontic measurements provides a quick way of assigning treatment type regarding extractions in Class I malocclusions.
